# Host-Adaptive Divergence Shapes the Genetic Architecture of *Magnaporthe oryzae* in Southern China’s Rice Agroecosystems

**DOI:** 10.3390/jof11070485

**Published:** 2025-06-26

**Authors:** Xin Liu, Jun Fu, Zhao Deng, Xinwei Chen, Xiaochun Hu, Zhouyi Tu, Qiuyi Wang, Yuxuan Zhu, Pengcheng Chen, Zhenan Bai, Tiangang Liu, Xuanwen Zhang, Peng Qin, Kai Wang, Nan Jiang, Yuanzhu Yang

**Affiliations:** 1 Key Laboratory of Southern Rice Innovation & Improvement, Ministry of Agriculture and Rural Affairs, Yuan Longping High-Tech Agriculture Co., Ltd., Changsha 410128, China13939861748@163.com (X.C.); tuzhouyi@lpht.com.cn (Z.T.);; 2 Hunan Engineering Laboratory of Disease and Pest Resistant Rice Breeding, Yuan Longping High-Tech Agriculture Co., Ltd., Changsha 410128, China; 3 State Key Laboratory of Hybrid Rice, Hunan Hybrid Rice Research Center, Changsha 410125, China; 4 Yuelushan Laboratory, Changsha 410128, China; 5 College of Life Sciences, Hunan Normal University, Changsha 410081, China; 6 College of Plant Science and Technology, Huazhong Agricultural University, Wuhan 430070, China

**Keywords:** *Magnaporthe oryzae*, rice blast, SNP, genetic diversity, population structure, mating type

## Abstract

Rice blast disease, caused by the ascomycete fungus *Magnaporthe oryzae* (syn. *Pyricularia oryzae*), poses a severe threat to global rice production. Southern China, a major rice-growing region characterized by diverse agroecological conditions, faces substantial challenges from blast disease, yet our understanding of the genetic structure of *M. oryzae* populations in this region remains limited. Here, we analyzed 885 *M. oryzae* strains from 18 nurseries across four rice ecological regions in Southern China using a panel of genome-wide SNP markers. Phylogenetic and principal component analyses revealed three distinct clonal lineages: lineage I (58.19%), lineage II (21.36%), and lineage III (20.45%). Lineage I exhibited a broader geographic distribution compared to the other two lineages. Host-adapted divergence was observed across rice subspecies, with lineage III predominantly associated with *japonica* growing-regions, while lineages I and II mainly colonized *indica* rice-growing regions. Genetic diversity exhibited significant spatial heterogeneity, with the nucleotide diversity (π) ranging from 0.17 in South China to 0.32 in the Middle–Lower Yangtze River region, reflecting differential cropping systems. The predominantly negative Tajima’s *D* values across populations suggested recent expansion or selective sweeps, likely driven by host resistance pressures. High genetic differentiation between lineage I and other lineages contrasted with low divergence between lineages II and III, indicating distinct evolutionary trajectories. Furthermore, an uneven distribution of mating types among three genetic lineages was observed, suggesting limited sexual recombination within clonal lineages. The information obtained in this study may be beneficial in devising suitable strategies to control rice blast disease in Southern China.

## 1. Introduction

As the cornerstone of global food security, rice (*Oryza sativa* L.) constitutes a vital dietary staple for over 3.5 billion people, especially in Asia [1]. With the global population projected to reach 9.7 billion by 2050, rice production must increase by at least 28% to meet escalating demands [2]. This challenge is further exacerbated by diverse stresses, among which rice diseases pose a particularly persistent threat [3]. Rice blast disease, caused by the ascomycete fungus *Magnaporthe oryzae* (syn. *Pyricularia oryzae*), stands as the most devastating threat. Annually, rice blast destroys 10–30% of the global rice yields, equivalent to losses sufficient to feed 60 million people, with economic damages exceeding USD 66 billion [4,5]. The pathogen’s plasticity and rapid evolution enable it to overcome host resistance (*R*) genes within 3–5 years, rendering even newly developed cultivars susceptible [6,7].

The rice—*M. oryzae* pathosystem has become a prime model for plant—pathogen research due to its economic importance and experimental tractability [8]. Beyond its role as a model organism, *M. oryzae* exhibits alarming ecological plasticity. It is capable of infecting over 50 monocot species, including economically and agriculturally important crop species such as wheat (*Triticum aestivum*), maize (*Zea maydis*), barley (*Hordeum vulgare*), foxtail millet (*Setaria italica*), and finger millet (*Eleusine coracana*), as well as wild grasses such as weeping lovegrass (*Eragrostis curvula*), ryegrass (*Lolium perenne*), and goosegrass (*Eleusine indica*) [9,10]. It is most well known as the agent of the ancient rice blast disease and the recently emerged wheat blast disease [6].

Notably, the avirulence (*AVR*) gene loci undergo accelerated diversification in response to selective pressures imposed by rice *R* genes. Population genetic analyses of *M. oryzae* allow us to investigate its genetic variability across different geographical locations, time periods, and host species, as well as the structure and differentiation of populations, providing insights into the pathogen’s evolutionary history and future evolutionary potential. Previous studies have indicated that *M. oryzae*’s wide host range is associated with intraspecific diversity [11,12,13,14,15]. Population genomic studies have revealed that *M. oryzae* populations are structured into host-specialized lineages [13]. Despite this host specialization, host jumps remain a critical concern for this pathogen. Wheat blast was first documented in the Paraná state of Brazil in 1985 [16]. Inoue et al. demonstrated that it moved to wheat likely from a local grass through a host jump [14].

The rice-infecting strains belong to a single genetic lineage (*Oryzae* lineage), and Southeast Asia is regarded as the key area regarding the origin, diversity, and dispersion of this lineage [12,13,17,18]. Rice blast disease is believed to have emerged as a result of a host jump from foxtail millet approximately 2500 to 7500 years ago [12]. Furthermore, the rice-infecting lineage at the global scale is subdivided into four lineages, which include three clonal lineages and one recombining lineage with more genetic diversity [13,19,20,21]. The recombining lineage, mainly distributed in Asia, shows a more balanced ratio of mating types, with higher female fertility rates. Female sterility, early post-mating genetic incompatibilities, and niche separation are considered to be responsible for the coexistence of the three clonal lineages [20]. These rice-infecting lineages were estimated to have diverged around 200~1000 years ago, long after rice domestication [13,19,21].

Southern China encompasses four rice ecological regions, namely the upper reaches of the Yangtze River (URYR), the middle and lower reaches of the Yangtze River (MLRYR), the Wuling Mountainous Area (WMA), and South China (SC), and accounts for 94% of the national planting area and 88% of the total output [22]. Diverse cropping systems and various groups of rice are observed in this region. In China’s major rice-growing regions, distinct cultivation patterns are observed based on the ecological conditions. URYR predominantly cultivates single-cropping *indica* rice, with *japonica* varieties restricted to higher-altitude areas. MLRYR demonstrates a mixed cultivation system, combining both double- and single-cropping indica rice, while japonica varieties are primarily cultivated in Jiangsu Province. WMA is characterized by single-cropping *indica* rice as the dominant variety, with *japonica* rice grown in limited quantities at higher elevations. SC maintains a consistent double-cropping system exclusively for *indica* rice varieties [23]. Its subtropical or tropical humid climates are extremely conducive to blast epidemics. Multiple reports have documented the genetic diversity and population structure of *M. oryzae* in various regions of Southern China [24,25,26,27,28,29]. However, these efforts face critical limitations in sampling, genotyping, and bioinformatic analysis, and the spatial dynamics of *M. oryzae* populations, as well as their adaptation to different agroecosystems, remain poorly understood. To fill these information gaps, we developed a panel of 146 SNP markers and constructed DNA fingerprints of large-scale *M. oryzae* strains across Southern China’s major ecological regions. We investigated the population structures and genetic diversity of these *M. oryzae* strains. The distribution of mating types was also examined. This work contributes to both a fundamental understanding of pathogen evolution and practical solutions for sustainable blast management in one of the world’s most critical rice-producing regions.

## 2. Materials and Methods

***M. oryzae* strain isolation, culture, and DNA extraction.** Rice blast-infected samples were collected from 18 disease nurseries between 2021 and 2022 (Table S1). The conidia of *M. oryzae* were blown onto the surface of a 2% water agar medium with an ear-washing bulb. After 2–4 h incubation at 28 °C, single spores were picked up under a stereomicroscope using a glass needle and transferred to a rice bran medium (40 g/L rice bran, 20 g/L agar, pH = 6.0–6.5). About 200 single spore strains were isolated for each blast nursery. The purified mycelia from single spores were preserved on sterilized filter paper discs under −20 °C. For DNA isolation, the filter paper discs were cultured on rice bran medium and grown at 28 °C for 7–8 days. Mycelium pieces of 7–15 mm in diameter and 100–300 mg in weight were harvested and transferred to a 2.0 mL centrifuge tube containing 0.8 mL of lysis buffer (200 mM Tris-HCl pH = 8.0, 50 mM EDTA, and 1% N-lauroylsarcosine). The mycelia were dispersed with a toothpick and incubated at room temperature for 15 min. The DNA was precipitated by adding absolute ethanol, followed by washing with 75% ethanol. The DNA pellet was air-dried and resolved in 0.05 mL TE buffer (pH = 8.0).

**Genome resequencing and SNP calling.** DNA libraries were prepared using the MGI DNA Library Prep Kit V4.0 (MGI Tech Co., Ltd., Shenzhen, China) and sequenced on a DNBSEQ-T7 platform (MGI Tech Co., Ltd., Shenzhen, China). In detail, DNA samples were fragmented, end-repaired, A-tailed, and ligated to MGI DNA adapters. The ligated DNA fragments were amplified by PCR and purified using VAHTS DNA Clean Beads (Vazyme, Nanjing, China). The PCR product concentration and fragment size distribution were assessed using a Qubit instrument (Thermo Fisher Scientific, Waltham, MA, USA) and an Agilent 2100 Bioanalyzer instrument (Agilent Technologies, Santa Clara, CA, USA), respectively. Libraries were then subjected to single-strand circularization using DNA circularization reagents. The resulting circular ssDNA was used to prepare DNA nanoballs (DNBs) by rolling circle amplification (RCA). The DNBs were loaded onto patterned nanoarray flow cells and sequenced on the DNBSEQ-T7 platform using the DNBSEQ-T7 high-throughput sequencing set. Sequencing was performed in paired-end mode with a read length of 150 bp. Sequencing data were subjected to quality control using fastp (v0.23.2) [30]. The cleaned reads were aligned to the *M. oryzae* MG8 reference genome (Ensembl release 53) using the bwa mem algorithm (v2.2.1) [31]. PCR and optical duplicates were marked using Picard tools (v2.23.0). SNP and InDel variants were called using GATK Haplotype Caller (v4.1.7.0) [32]. Gene-based variant annotation was conducted using SnpEff (v4.3t) [33].

**KASP marker design and genotyping.** To ensure high-quality SNPs for KASP marker design, variants were filtered based on standard GATK hard-filtering recommendations. “https://gatk.broadinstitute.org/hc/en-us/articles/360035890471-Hard-filtering-germline-short-variants“ (accessed on 27 April 2024). We removed SNPs with any of the following characteristics: low variant call quality (QUAL < 30.0), low quality by depth (QD < 2.0), high strand bias (SOR > 3.0 or FS > 60.0), poor read mapping quality (MQ < 40.0), or significant biases in mapping quality or read position between reference and alternative alleles (MQRankSum < −12.5 or ReadPosRankSum < −8.0). These criteria help to eliminate potential artifacts from sequencing and alignment. Additionally, we filtered out SNPs with missing data in more than one sample, SNPs located within 20 bp of InDels, and non-biallelic SNPs and retained only SNPs where at least one sample was a homozygous reference (0/0) and one sample was a homozygous alternative (1/1). Using bcftools [34], we randomly selected one qualifying polymorphic SNP per 100 kb window. We developed KASP assays by selecting SNPs with high polymorphic potential and strategic chromosomal locations. Optimized marker design was ensured using the BatchPrimer3 (v1.0) software, and primer specificity was confirmed with BLAST (v2.10.1). The KASP assay was performed on a high-throughput IntelliQube genotyping platform (LGC Genomics Ltd., Hoddesdon, UK) with a reaction volume of 1.6 µL, comprising 0.8 µL (15–30 ng) DNA template, a 0.8 µL mixture of 2×Master mix, and 0.07 μL primer mix. The KASP assay utilized a touchdown PCR method: 94 °C for 15 min; 94 °C for 20 s and 61 °C for 60 s (decreasing 0.6 °C per cycle) for 10 cycles; and 94 °C for 20 s and 55 °C for 60 s for 26 cycles. The fluorescence signals of the amplification products were detected and classified.

**Population structure and genetic diversity analysis.** To construct a phylogenetic tree, the pairwise identity-by-descent (IBD) distance matrix was first calculated using PLINK (v1.9) [35]. Then, a neighbor-joining tree was constructed using the R package APE (v5.4) [36]. Principal component analysis (PCA) was performed using the R package smartpca (v7.2.1) to visualize the genetic relationships among individuals [37]. The top three principal components, which explained the largest proportion of the variance, were plotted. DNA sequence diversity indices, including nucleotide diversity (π) and Tajima’s *D*, were calculated for each chromosome using VCFtools (v0.1.16) [34]. The pairwise fixation index (*F*_ST_) was calculated for each chromosome as a window. The mean *F*_ST_ values across all seven chromosomes were considered as the final results. Data visualization was performed using the Matplotlib library (v3.6.2) in Python (v3.10.16).

## 3. Results

**Figure 1:**
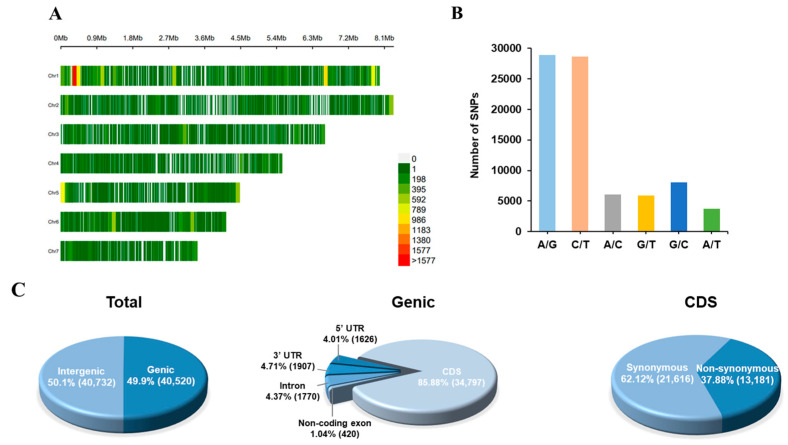
Summary of SNPs of mixed *M. oryzae* strains from 8 nurseries in Southern China. (**A**) Distribution of SNPs across seven *M. oryzae* chromosomes. (**B**) Number of different substitution types in the identified SNPs. (**C**) Annotation of SNPs.

**Genome resequencing and SNP identification.** In order to develop genome-wide SNP markers suitable for the genotyping of *M. oryzae* populations in Southern China, fifty *M. oryzae* strains from each of the eight nurseries (across four rice ecological regions) were pooled into separate DNA samples for whole-genome sequencing. Each of the eight pooled samples generated 9.77–13.28 Gb of raw data (Table S2). After cleaning, 9.63~13.09 Gb high-quality data remained. The average mapping rate of the sequencing reads to the reference genome of the laboratory strain 70–15 was approximately 90.40%, and the average genome coverage depth was approximately 204.24×. A total of 157,024 raw SNPs were detected and 81,252 high-quality SNPs were obtained after filtering. The SNP density varied across different chromosomes, with the highest and lowest SNP densities detected on chromosome 1 (3.38 SNP/kb) and chromosome 4 (1.42 SNP/kb), respectively (Figure 1A; Table S3). Among these SNPs, the proportion of transitions (A/G or C/T) was significantly higher than the proportion of transversions (A/C, G/T, G/C, or A/T) (Figure 1B; Table S4). The ratio of transitions (57,499) to transversions (23,753) was 2.42. Nearly half of the SNPs were distributed in intergenic regions (40,732), while the other half were in genic regions (40,520) (Figure 1C). The majority of genic SNPs were distributed in the CDS region (34,797), followed by the 3′ UTR (1907), intron (1770), 5′UTR (1626), and non-coding exon (420) regions. Of the 34,797 SNPs in the CDS region, 37.9% and 62.1% were nonsynonymous and synonymous, respectively.

**Figure 2:**
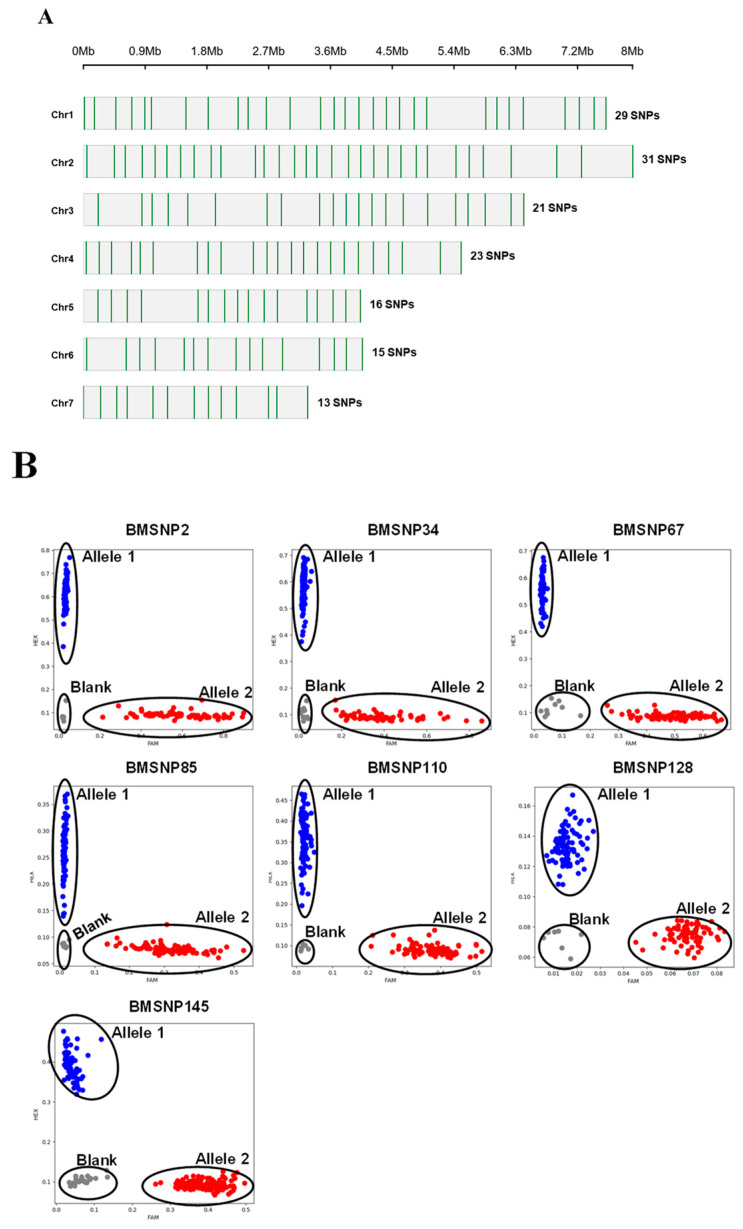
Development of KASP markers for *M. oryzae* genotyping. (**A**) Distribution of KASP markers across seven *M. oryzae* chromosomes. The green vertical lines indicate the SNP positions on the chromosomes. (**B**) Genotyping patterns of seven representative markers based on KASP assay. Scatter plot shows FAM fluorescence (X-axis) versus HEX fluorescence (Y-axis). Blank represents negative control.

**Development of KASP markers.** To facilitate SNP-based genotyping, a total of 384 SNPs were obtained, evenly distributed throughout the whole genome, as described in the Materials and Methods. Subsequently, 204 candidate SNPs were successfully designed for KASP markers, which were comprehensively screened with the 80 *M. oryzae* strains from 8 nurseries. Based on the discrimination ability, stability, and relatively uniform distribution of SNP loci, 146 high-quality KASP markers were selected for *M. oryzae* genotyping (Table S5; Figure 2A,B). The number of KASP markers per chromosome ranged from 13 on chromosome 7 to 31 on chromosome 2. The average marker density was 3.51 Mb across the entire genome, ranging from 2.88 Mb on chromosome 1 to 4.15 Mb on chromosome 4.

**Figure 3:**
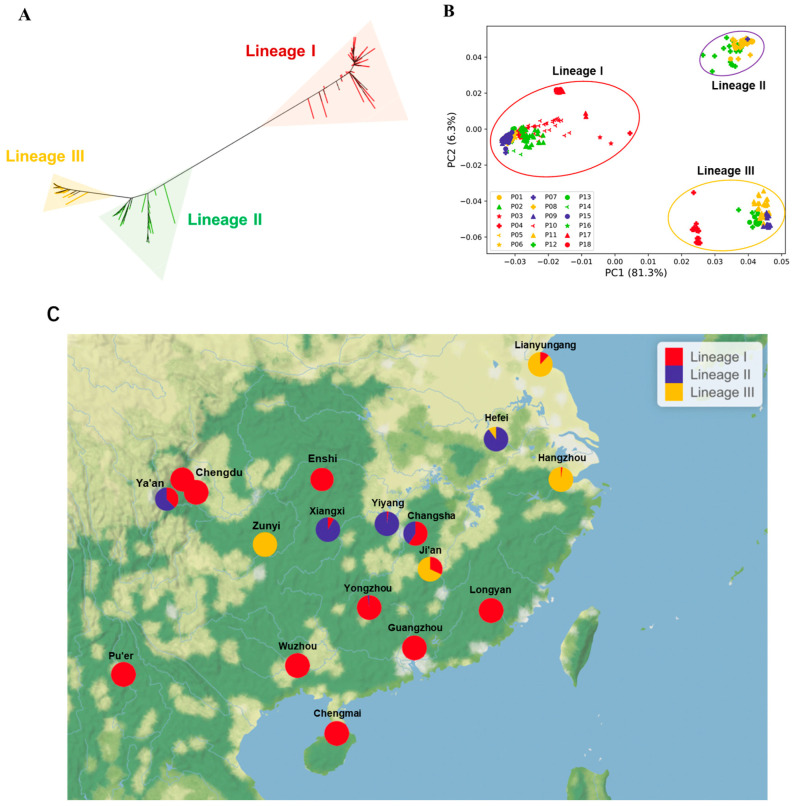
Phylogeny and population structure of *M. oryzae* strains in Southern China. (**A**) Phylogenetic tree constructed using the neighbor-joining method based on 885 strains. (**B**) Principal component analysis (PCA) of three lineages. P01–P18 represent 18 different nurseries. P01, Yucheng, Ya’an, Sichuan; P02, Pujiang, Chengdu, Sichuan; P03, Qionglai, Chengdu, Sichuan; P04, Meitan, Zunyi, Guizhou; P05, Taojiang, Yiyang, Hunan; P06, Fenghuang, Xiangxi, Hunan; P07, Jianghua, Yongzhou, Hunan; P08, Liuyang, Changsha, Hunan; P09, Jinggangshan, Ji’an, Jiangxi; P10, Xuan’en, Enshi, Hubei; P11, Lin’an, Hangzhou, Zhejiang; P12, Hefei, Anhui; P13, Ganyu, Lianyungang, Jiangsu; P14, Shanghang, Longyan, Fujian; P15, Conghua, Guangzhou, Guangdong; P16, Cenxi, Wuzhou, Guangxi; P17, Chengmai, Hainan; P18, Pu’er, Yunnan. (**C**) The distribution and composition of the three lineages within 18 Southern China nurseries. There are two nurseries in Chengdu, located in Pujiang and Qionglai, respectively. Beyond Chengmai County, which directly belongs to Hainan Province, other places are elevated to the city level.

**Phylogeny and population structure of *M. oryzae* strains in Southern China.** We genotyped 885 *M. oryzae* strains from 18 blast nurseries across Southern China with the panel of 146 KASP markers. A neighbor-joining analysis was performed and these strains were classified into three lineages: lineage I (515 strains, 51.89%), lineage II (189 strains, 21.36%), and lineage III (181 strains, 20.45%) (Figure 3A). To further validate this categorization, we conducted PCA. Consistent with the phylogenetic results, the PCA also categorized the strains into three lineages (Figure 3B). The first and second PCs, explaining 81.3% and 6.30% of the total variance, respectively, indicated the possible separation of the three lineages (Figure 3B). Lineage I is widely distributed across Southern China, whereas lineage II and lineage III are found in the Yangtze River Basin and Wuling Mountainous Area (Figure 3C). It seems that strains from lineage III dominate in regions that are associated with the cultivation of *japonica* varieties, whereas lineage I and lineage II dominate in regions where *indica* rice varieties are prevalent.

**Figure 4:**
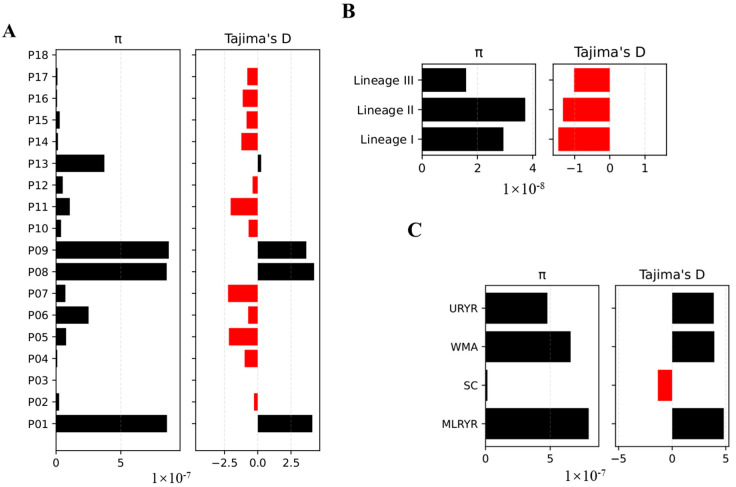

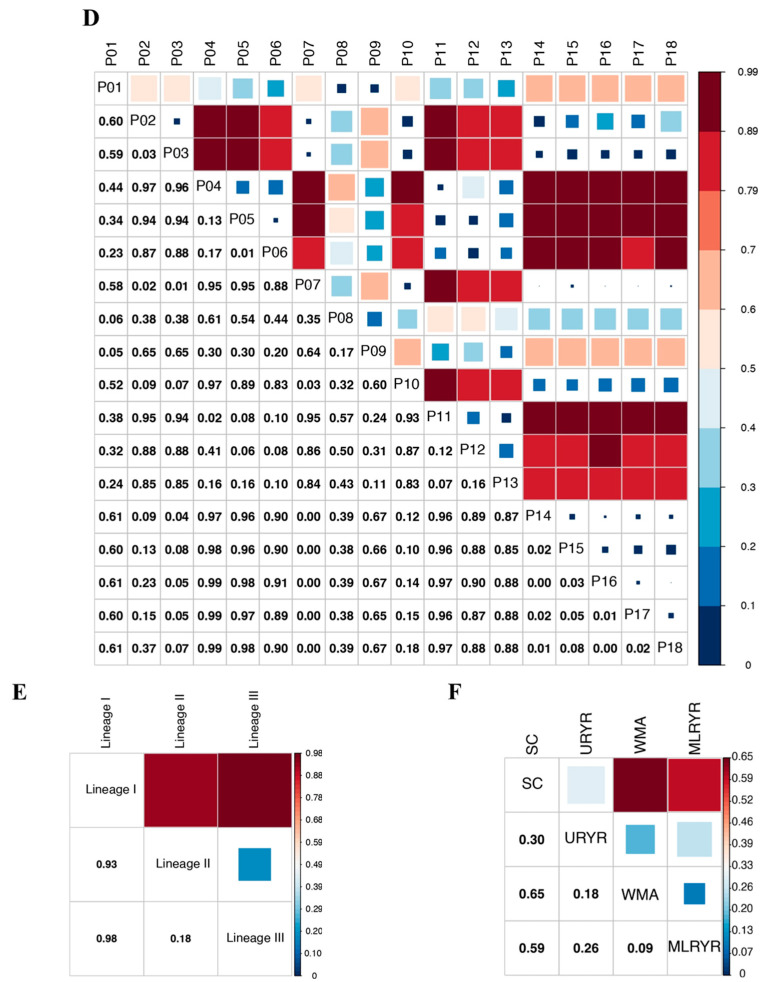
Genetic diversity analysis of field *M. oryzae* strains in Southern China. Nucleotide diversity (π) and Tajima’s *D* values of nurseries (**A**), lineages (**B**), and ecological regions (**C**). Red indicates negative values, suggesting possible selective pressure, while black represents positive values. Fixation index (*F*_st_) values of nurseries (**D**), lineages (**E**), and ecological regions (**F**). P01–P18 represent 18 different nurseries. P01, Yucheng, Ya’an, Sichuan; P02, Pujiang, Chengdu, Sichuan; P03, Qionglai, Chengdu, Sichuan; P04, Meitan, Zunyi, Guizhou; P05, Taojiang, Yiyang, Hunan; P06, Fenghuang, Xiangxi, Hunan; P07, Jianghua, Yongzhou, Hunan; P08, Liuyang, Changsha, Hunan; P09, Jinggangshan, Ji’an, Jiangxi; P10, Xuan’en, Enshi, Hubei; P11, Lin’an, Hangzhou, Zhejiang; P12, Hefei, Anhui; P13, Ganyu, Lianyungang, Jiangsu; P14, Shanghang, Longyan, Fujian; P15, Conghua, Guangzhou, Guangdong; P16, Cenxi, Wuzhou, Guangxi; P17, Chengmai, Hainan; P18, Pu’er, Yunnan. Abbreviations: URYR, upper reaches of the Yangtze River; WMA, Wuling Mountainous Area; SC, South China; MLRYR, middle and lower reaches of the Yangtze River.

**Genetic diversity of the *M. oryzae* population.** To assess the genetic diversity within the populations of *M. oryzae*, we conducted analyses of the nucleotide diversity (π), Tajima’s *D*, and fixation index (*F*_ST_) based on the nurseries, ecological regions, and lineages. The π values displayed significant variation across sampling locations, with four nurseries (Ya’an-P01, Changsha-P08, Ji’an-P09, and Lianyungang-P13) demonstrating particularly high levels (Figure 4A). When comparing the four ecological regions, MLRYR showed the highest nucleotide diversity, whereas SC displayed the lowest (Figure 4C). Across the three lineages, lineage II exhibited the highest nucleotide diversity, whereas lineage III had the lowest (Figure 4B). Tajima’s *D* was predominantly negative in most nurseries, all lineages, and South China, indicating an excess of low-frequency polymorphisms (Figure 4A–C). The *F*_ST_ values among various nurseries ranged widely from 0.00 to 0.99, with the lowest *F*_ST_ values observed between P07 and P14, P07 and P15, P07 and P16, P07 and P17, P07 and P18, P16 and P14, and P16 and P18, suggesting a high degree of relatedness among strains from these nurseries (Figure 4D). Lineage I was markedly differentiated from lineage II and lineage III, with *F*_ST_ values of 0.93 and 0.98, respectively (Figure 4E). Conversely, lineage II and lineage III were more closely related, with a lower *F*_ST_ value of 0.18. Among the ecological regions, SC was highly differentiated from both WMA and MLRYR, whereas WMA and MLRYR were more closely related (Figure 4F).

**Mating type distribution. ***M. oryzae* comprises two distinct mating types (MAT1-1 and MAT1-2), which are determined by a single locus on chromosome 7 [38]. Parasexual reproduction requires two strains of opposite mating types, with at least one being female-fertile and capable of producing perithecia [39]. The mating type alleles have been used as markers to investigate population diversity in the pathogen [40,41]. In this study, a KASP marker at the MAT locus was developed for the rapid determination of the mating types of 370 *M. oryzae* strains from nine nurseries across four rice ecological regions (Table S6). In total, 166 (44.9%) strains were MAT1-1 and 195 (52.7%) strains were MAT1-2, indicating that the distribution of MAT1-1 and MAT1-2 is relatively balanced (Table S7). Additionally, both mating types were detected simultaneously in the nine (2.4%) strains. However, the mating types were unevenly distributed among three lineages: 97.5% of lineage I strains were MAT1-2, while 97.8% of lineage II strains and 97.0% of lineage III strains were MAT1-1.

## 4. Discussion

Over the past two decades, population-genetic studies of *M. oryzae* have yielded several critical findings: (1) Southeast Asia is recognized as both the center of origin and a hotspot for the genetic diversity of this pathogen; (2) global populations are composed of four genetic lineages—one recombining lineage primarily found in Southeast Asia and three clonal lineages with broad geographical distributions; (3) the recombining lineage displays balanced mating types and frequent sexual fertility, contrasting with clonal lineages that exhibit skewed mating type ratios and rare sexual reproduction; (4) these lineages exhibit host specialization toward major rice groups. In this study, genome-wide SNP analyses of *M. oryzae* populations in Southern China revealed three lineages with imbalanced mating type ratios, consistent with global clonal lineage signatures. The absence of recombining lineages in our study may stem from sampling limitations—only one site in Yunnan (the primary distribution region of recombinant lineage strains in China) and an exclusive focus on modern cultivars displaying relatively low genetic diversity in terms of genetic background. Lineage I, as the dominant genetic lineage, showed a broad distribution across all ecological regions, while lineages II and III were restricted to the Yangtze River Basin and Wuling Mountainous Area. Lineage III dominated *japonica*-growing regions, whereas lineages I and II were primarily distributed in *indica* cultivation regions, suggesting host-adapted divergence across rice subspecies. The observed pattern aligns with previous findings demonstrating lineage-specific host preferences in *M. oryzae* populations [13,20,21]. However, this host specialization is not strict, suggesting additional ecological drivers beyond subspecies specificity. Notably, a recent work reveals differential geographic ranges among *M. oryzae* lineages [20]. This ecological dimension becomes particularly relevant given the well-established agroecological differentiation between *indica* and *japonica* subspecies—where *indica* cultivars dominate tropical/lowland cultivation systems, while *japonica* cultivars prevail in temperate/high-altitude regions [42,43]. The apparent congruence between the lineage distributions and their host cultivation ecologies raises critical questions about the potential covariation of host adaptation and environmental adaptation in this pathosystem. Future studies should therefore employ integrated approaches to disentangle the relative contributions of host-specific selection versus abiotic environmental factors in driving *M. oryzae* population differentiation.

Genetic diversity analyses revealed significant heterogeneity across nurseries, ecological regions, and lineages. In particular, MLRYR displayed high nucleotide diversity (π = 0.32). This rice ecological region is characterized by differential rice cropping systems and the mixed cultivation of *indica* and *japonica* cultivars, both of which could foster genetic exchange and diversification. Conversely, South China (SC) displayed the lowest π value among the ecological regions, possibly due to monoculture practices, uniform host genotypes, or stronger directional selection from widespread resistance genes. The predominantly negative Tajima’s *D* values across nurseries and lineages indicate an excess of low-frequency polymorphisms, a signature consistent with recent population expansion or selective sweeps. This pattern aligns with the rapid adaptation of *M. oryzae* to resistant rice varieties, where selective pressures drive the fixation of advantageous alleles and purge genetic diversity at linked loci [21,44]. For example, lineage III’s low π and strong negative Tajima’s *D* may reflect a recent clonal expansion following the adoption of *japonica*, leading to selective sweeps at effector loci. The high genetic differentiation between lineage I and lineages II/III (*F*_ST_ = 0.93–0.98) underscores their distinct evolutionary trajectories. By contrast, the lower *F*_ST_ between lineages II and III (0.18) suggests recent divergence or occasional gene flow, potentially facilitated by overlapping host ranges or environmental niches. It is worth noting that these two genetically closely related lineages display different host specificities. This may be somewhat associated with the current rice breeding strategies and agricultural practices. The increasing cultivation and application of *indica*–*japonica* hybrid rice cultivars and *indica* hybrid rice cultivars with introgressed *japonica* genomes may play a role in promoting the differentiation of lineage III.

The mating type analysis revealed a near-equal distribution of MAT1-1 (44.9%) and MAT1-2 (52.7%) alleles across the 370 tested strains. However, this balance was not uniform across lineages: lineage I was overwhelmingly MAT1-2 (97.5%), lineage II predominantly MAT1-1 (97.8%), and lineage III mostly MAT1-1 (97.0%). Such skewed ratios suggest that sexual reproduction is rare or absent within lineages, favoring clonal propagation. This is consistent with earlier findings in rice-infecting clonal lineages, as well as wheat blast populations, where clonal expansion driven by agricultural practices has led to mating type imbalance [19,45].

In this study, by leveraging genome-wide SNP markers and extensive sampling, we uncovered three distinct lineages of *M. oryzae* with differential geographic and host-associated distributions, uneven genetic diversity across ecological regions, and an imbalanced mating type ratio among lineages. These findings offer novel insights into the pathogen’s evolutionary trajectory and practical implications for blast disease management. Future work should expand genomic surveillance across spatiotemporal scales and integrate functional genomics to unravel the molecular basis of host adaptation.

## 5. Conclusions

This study provides a comprehensive analysis of the genetic architecture and population dynamics of *Magnaporthe oryzae* in Southern China’s rice agroecosystems, revealing three distinct clonal lineages with host-adapted divergence. Lineage I, the most prevalent, exhibited a broad geographic distribution, while lineages II and III showed niche specificity, particularly in *indica*- and *japonica*-growing regions, respectively. Significant spatial heterogeneity in genetic diversity, as evidenced by the nucleotide variation and Tajima’s *D* values, suggests recent population expansion or selective sweeps driven by host resistance pressures. The high genetic differentiation between lineage I and the other lineages underscores their divergent evolutionary trajectories, whereas the closer relationship between lineages II and III hints at potential gene flow or shared ancestry. Furthermore, the skewed distribution of mating types within lineages indicates limited sexual recombination, favoring clonal propagation. These findings highlight the importance of host specialization and ecological factors in shaping pathogen evolution. The insights gained from this study can inform targeted disease management strategies, such as the deployment of resistant rice varieties tailored to regional pathogen populations, and they underscore the need for continuous genomic surveillance to monitor emerging pathogen adaptations in this critical rice-producing region.

## Data Availability

The resequencing data of *Magnaporthe oryzae* have been deposited in the NCBI Sequence Read Archive (SRA) under the BioProject accession number PRJNA1274526.

## Supplementary Materials

The following supporting information can be downloaded at: https://www.mdpi.com/article/10.3390/jof11070485/s1, Table S1: The information of *M. oryzae* strains used in this study. Table S2: Statistics of re-sequencing data of mixed *M. oryzae* strains collected from eight nurseries in southern China. Table S3: Summary of chromosomal SNP distribution. Table S4: Number of different substitution types in the identified SNPs. Table S5: Information of KASP markers for *M. oryzae* genotyping. Table S6: Information of KASP marker for determining mating types of *M. oryzae*. Table S7: Mating type distribution of *M. oryzae* strains.
